# Notes and News

**Published:** 1895-05-18

**Authors:** 


### Notes and News.

(Collected and Communicated.)

The temptation which the possible life insurance of infants affords to unprincipled parents has been so fully recognised in America that steps are being taken in Massachusetts to prohibit the insurance of any child under ten years of age. We have only to watch a few cases in the newspapers, and observe how large a number of the infants who die from neglect in our cities especially have been insured by their parents, to feel that some restriction should be placed upon such proceedings in England also.

A dental hospital at Newcastle was opened last month by the Mayor of that town. The foundation-stone of the new infirmary at Bridgnorth was laid on the 24th ult. Mr. and Mrs. Foster, of Apley Park, contributed £1,000 towards the building, whilst a bequest of £7,000 from a lady in the neighbourhood added £5,000 towards the endowment fund and £2,000 towards the building. The whole cost of the building, amounting to upwards of £4,300, has now been raised. Provision is made for 18 beds, the wards being arranged to obtain a western exposure. The foundation-stone of a cottage hospital for Bridgend, Wales, has just been laid. The estimated cost, amounting to a sum of £1,500, has mainly been collected by the proceeds of a yearly Eisteddfod at Bridgend. A sum of £300 is still required.

The Great Ormond Street Children's Hospital is much in need of funds this year, and the Committee have been appealing for 100 ladies to help in collecting sums of £5 and upwards to be placed upon the Duke of Cambridge's list at the annual dinner on May 17th. The festival last year resulted in the splendid sum of £11,000, and it was, therefore, hardly to be expected that a like amount would be realised on the present occasion, but so far only £1,000 has been collected, whilst at least £4,000 is urgently required to carry on the good work done within the walls of this institution. We hope Friday's dinner will see the announcement of a sum adequate to the needs of the hospital, and that a charity of such proved usefulness will not be allowed by a generous public to suffer for want of deserved support.

An exhibition of medicinal and useful plants will be held at the Hague in July. Intending exhibitors must apply before June 15th to Dr. Gresshof, 97, Laan van Meerdevoot, The Hague.

The Princess of Wales, when she opens the grand bazaar in aid of St. Mary's Hospital at the Portman Rooms on June 27th, will be received on her arrival by the Duke and Duchess of York.

On Saturday, May 11th, the Caxton Wing of the Morley House Convalescent Home, St. Margaret's, was opened by the Hon. W. F. D. Smith, M.P., accompanied by Lady Esther Smith.

A festival dinner in aid of the funds of the East London Hospital for Children, Shadwell, is announced to take place on May 30th, at the Hall of the Worshipful Company of Leathersellers. The Earl of Errol will be in the chair.

There is no Guy's man living who has not grieved over the death of Mr. Arthur E. Durham. He was a favourite pupil of Hilton, an able anatomist, a skilful surgeon, a most amusing and instructive teacher, and the best of good fellows. Arthur Durham was always a popular personage, and the kindness and tenderness of his heart made him beloved by all who came within the sphere of his influence. Good comrade, good surgeon, good teacher, and good friend, Arthur Durham's memory will linger pleasantly in the minds of all who knew him.

Quite a new departure has been made in hospital meetings at Wolverhampton. At the last annual meeting of the District Hospital for Women, "the business proceedings (to use the words of the local reporter) were blended with musical contributions of one kind or another." "One half of the great hall, screened off and curtained, had been transformed into an Oriental drawing-room, whilst a kind of five o'clock tea, so dear to the feminine heart, added to the enjoyment of the whole." The meeting seems to have been very largely attended, and the proceedings regarded as highly successful.

Many secretaries are of opinion that benefit performances for hospitals cost in the long run more than they yield to a charity. The concert held on the 17th inst., in aid of the Royal Hospital for Diseases of the Chest, seems to be a case in point, as the secretary issued an urgent whip, to the effect "that the sale of tickets had been far from satisfactory," and urging each Governor to purchase some tickets, as two well known members of the Council had guaranteed the expenses, £200. A circular like this is calculated to upset many Governors, and, in our experience, to do more harm than good. It would be helpful to other secretaries if Mr. Harrold would tell us its effect in the present instance, and how far it tended to retrieve the success of the concert in question. Unfortunately, there are too many people in this world who, if they buy a ticket for a concert, will advance that as a proof of their great interest in a particular charity, and express surprise that they should be worried further for subscriptions.

